# Identification of PDCD1 as a potential biomarker in acute rejection after kidney transplantation *via* comprehensive bioinformatic analysis

**DOI:** 10.3389/fimmu.2022.1076546

**Published:** 2023-01-27

**Authors:** Yucheng Wang, Xiaoli Lin, Cuili Wang, Xinyu Liu, Xiaoying Wu, Yingying Qiu, Ying Chen, Qin Zhou, Haige Zhao, Jianghua Chen, Hongfeng Huang

**Affiliations:** ^1^ Kidney Disease Center, The First Affiliated Hospital, College of Medicine, Zhejiang University, Hangzhou, Zhejiang, China; ^2^ Key Laboratory of Kidney Disease Prevention and Control Technology, Hangzhou, Zhejiang, China; ^3^ Institute of Nephrology, Zhejiang University, Hangzhou, Zhejiang, China; ^4^ Zhejiang Clinical Research Center of Kidney and Urinary System Disease, Zhejiang, China; ^5^ Department of Cardiothoracic Surgery, The First Affiliated Hospital, Zhejiang University School of Medicine, Hangzhou, China

**Keywords:** acute rejection, PDCD1, kidney transplantation, biomarker, immune infiltration, weighted gene co-expression network analysis, T cell receptor signaling pathway, T cell exhaustion

## Abstract

**Background:**

Acute rejection is a determinant of prognosis following kidney transplantation. It is essential to search for novel noninvasive biomarkers for early diagnosis and prompt treatment.

**Methods:**

Gene microarray data was downloaded from the Gene Expression Omnibus (GEO) expression profile database and the intersected differentially expressed genes (DEGs) was calculated. We conducted the DEGs with Gene Ontology (GO) and Kyoto Encyclopedia of Genes and Genomes (KEGG) enrichment analysis. Distribution of immune cell infiltration was calculated by CIBERSORT. A hub gene marker was identified by intersecting the rejection-related genes from WGCNA and a selected KEGG pathway—T cell receptor signaling pathway (hsa04660), and building a protein-protein interaction network using the STRING database and Cytoscape software. We performed flow-cytometry analysis to validate the hub gene.

**Results:**

A total of 1450 integrated DEGs were obtained from five datasets (GSE1563, GSE174020, GSE98320, GSE36059, GSE25902). The GO, KEGG and immune infiltration analysis results showed that AR was mainly associated with T cell activation and various T-cell related pathways. Other immune cells, such as B cells, Macrophage and Dendritic cells were also associated with the progress. After utilizing the WGCNA and PPI network, PDCD1 was identified as the hub gene. The flow-cytometry analysis demonstrated that both in CD4^+^ and CD8^+^ T cells, PD1^+^CD57^-^, an exhausted T cell phenotype, were downregulated in the acute rejection whole blood samples.

**Conclusions:**

Our study illustrated that PDCD1 may be a candidate diagnostic biomarker for acute kidney transplant rejection *via* integrative bioinformatic analysis.

## 1 Introduction

Kidney transplantation is currently a primary treatment for patients with end-stage renal disease, with higher life-quality and less medical resource consumption compared with other renal replacement therapies (1). Despite the dramatic improvement of short-term outcomes over decades, long-term outcomes of kidney transplantation are still remaining an obstacle (2). Acute renal allograft rejection was noted as the leading primary or secondary cause of short-term and long-term graft failure (3). Prompt recognition and aggressive treatment for acute rejection (AR) are essential for preventing the progression of the disease. Except for renal biopsy, other monitoring measures of AR, such as renal function, donor-specific antibodies, and serum immunosuppressive drug level, are much less specific (4). Accurate noninvasive novel biomarkers have high negative predictive value and may substitute biopsy surveillance. Therefore, researchers are now paying attention to exploring ideal noninvasive novel biomarkers related to renal allograft rejection.

Previous studies have detected gene expression profiles through microarray measurements in peripheral blood, urine, and biopsy samples to differentiate rejection patients from non-rejection (5). In 2012, a five-gene set (DUSP1, PBEF1, PSEN1, MAPK9, and NKTR) was locked by qPCR and penalized logistic regression verification and validation in peripheral blood samples (6). Chen et al. identified and validated three cross-organ AR biomarkers (PECAM1, CXCL9, and CD44) by integrating three datasets of pediatric renal transplantation, adult renal transplantation and heart transplantation (7). Bioinformatic analyses of the updated datasets could aid in discovering novel candidate genes and provide deeper understandings of the molecular mechanisms of acute renal transplant rejection.

The development of new computational techniques remains a rapidly expanding area. Weighted gene co-expression network analysis (WGCNA) is a systems biology method to transform the gene expression data into highly relevant modules and investigate genes in the selected modules, which is beyond traditional differential expression detection (8). CIBERSORT, a deconvolution algorithm, could calculate the levels and examine the roles of immune cells in the immune microenvironment of the target disease (9). Currently, both methods have been applied effectively in various diseases to identify hub genes or therapeutic agents (10, 11), while studies using WGCNA or immune infiltration to search for the potential biomarkers in renal transplant AR are still scarce.

In this study, differentially expressed genes (DEG) between AR and non-rejection (NR) samples were identified from five gene expression omnibus (GEO) microarray datasets (GSE1563, GSE25902, GSE36059, GSE98320, and GSE174020). We conducted the WGCNA analysis, and two up-regulated modules, medium purple 3 and dark orange, and one down-regulated module, midnight blue, were chosen as rejection-related modules, as well as immune cell infiltration and protein-protein interaction (PPI) network analyses to screen PDCD1, a hub gene involved in kidney transplant rejection. Flow cytometry analyses with an exhaustion-related phenotype, PD1+CD57-, validated the identified hub gene.

## 2 Materials and methods

### 2.1 Gene expression profiles and ethical approval

Five datasets related to acute rejection, including GSE1563, GSE25902, GSE36059, GSE98320, and GSE174020, were obtained from the NCBI Gene Expression Omnibus (GEO) database (https://www.ncbi.nlm.nih.gov/geo/). Detailed information of the datasets is listed in **Table 1**. All datasets were from renal allograft biopsy-based microarray studies. Heatmaps of the datasets were generated by the ‘pheatmap’ R package. Informed consent was exempted as the study relied on past clinical sample collections, did not involve any identifying information and brought no harm to the subjects. This study was approved by the Ethics Committee of the First Affiliated Hospital, Zhejiang University School of Medicine (Reference Number: 2022-0857).

**Table 1:** The information of five kidney allograft acute-rejection related datasets from the GEO database.

GEO number [year]	Acute rejection	Non-rejection	Sample type	Platforms	Description
GSE1563 [2004]	7	10	renal biopsies	GPL8300	Affymetrix Human Genome U95 Version 2 Array
GSE25902 [2011]	24	96	renal biopsies	GPL570	Affymetrix Human Genome U133 Plus 2.0 Array
GSE36059 [2013]	100	281	renal biopsies	GPL570	Affymetrix Human Genome U133 Plus 2.0 Array
GSE98320 [2017]	434	774	renal biopsies	GPL15207	Affymetrix Human Gene Expression Array
GSE174020 [2021]	7	11	renal biopsies	GPL571	Affymetrix Human Genome U133A 2.0 Array

### 2.2 DEG screening and functional enrichment analysis

Differentially expressed genes (DEGs) were calculated using the GEO2R online tool (https://www.ncbi.nlm.nih.gov/geo/geo2r/) with the cut-off criteria of adjusted P. value<0.05. Intersection of the DEGs was visualized using Venn plot performed online (http://jvenn.toulouse.inra.fr/app/example.html). To investigate the function of the DEGs, Gene Ontology (GO) analysis, including biological processes (BP), cellular components (CC), and molecular functions (MF), and Kyoto Encyclopedia of Genes and Genomes (KEGG) pathway enrichment analysis were performed by the ‘clusterprofiler’ R package and plotted with the ‘ggplot2’ R package (12, 13). The cut-off of enrichment significance was P<0.05.

### 2.3 Analysis of immune cell infiltration

CIBERSORTx website (https://cibersortx.stanford.edu/) was used to calculate the abundance of 22 immune cell types (LM 22) in the GSE25902 dataset (14). Heatmaps of the immune cell abundance were performed by the ‘pheatmap’ R package. Comparison between the acute rejection group and the non-rejection group was visualized by the ‘ggplot2’ R package.

### 2.4 Construction of weighted correlation network analysis

Gene expression array from GSE25902 was selected to conduct WGCNA using the ‘WGCNA’ package in R (8). The main processes used in WGCNA were sample clustering to remove outlier samples, co-expression network construction and module identification, and identification of acute rejection-associated modules. Module- and phenotype-associated genes were screened under the following conditions: gene significance (GS) >0.2 and module membership (MM) > 0.7. Intersection of the correlated genes and genes recorded in the T cell receptor signaling pathway (hsa04660) was performed by Venn plot online. We used ‘pathview’ R package to mark the intersected genes in the pathway.

### 2.5 Protein-protein Interaction network construction and hub gene identification

We applied the STRING database (https://string-db.org/) (15) to construct the PPI network of the intersected DEGs. These intersected DEGs were imported into Cytoscape v3.9.1 (16) and were ranked using the DMNC and ClusteringCoefficient algorithms in Cytohubba to identify the hub gene.

### 2.6 Validation of hub gene with flow cytometric analysis

Whole blood samples from 126 healthy control, 146 pre-transplant recipients, 30 recipients with stable renal function (STA), and 12 recipients with AR were obtained from the First Affiliated Hospital, College of Medicine, Zhejiang University. The research protocol was approved by the Research Ethics Committee. White blood cell was purified using Red Blood Cell Lysis Buffer (Solarbio, R1010) twice and incubated with conjugated antibiotics including CD4-APC, CD8-PE-Cy™7, CD279 (PD1)-PE, CD57-FITC (BD bioscience, 663498, 335822, 560795, 663497) for 20 minutes at room temperature in dark. After washing, cells were resuspended in PBS. Data was acquired on FACSCanto II flow cytometer (BD Bioscience) and analyzed with FlowJo V10.

### 2.7 Statistical analysis

Statistical Analysis was performed using SPSS software (version 23; SPSS, Inc., Chicago, IL, United States). A two-sided P. value<0.05 is considered a significant difference.

## 3 Results

### 3.1 Identification and functional enrichment analysis of DEGs

**Figure 1:**
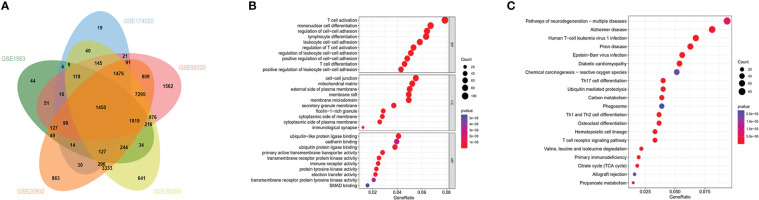
Functional enrichment analysis and Venn diagram of DEGs: **(A)** Venn diagram demonstrating DEGs in the 5 datasets (GSE1563, GSE174020, GSE98320, GSE36059, GSE25902). **(B)** GO analysis for DEGs. **(C)** KEGG analysis for DEGs.

We retrospectively analyzed five eligible GEO datasets (GSE1563, GSE25902, GSE36059, GSE98320, GSE174020) related to renal graft acute rejection. **Table 1** presented the detailed information of the selected datasets. We used the GEO2R online tool to screen genes with P-Values<0.05 and the top 50 DEGs for each dataset were presented in heatmaps (**Figure S1**). To explore the most valuable DEGs, we visualized the DEGs with Venn plot and obtained 1450 intersected DEGs (**Figure 1A**), which were then enriched by the ‘clusterprofiler’ R package. The top 10 significantly enriched biological processes, cellular components, and molecular function GO terms are shown in **Figure 1B**, and the top 20 significantly enriched KEGG terms are shown in **Figure 1C**. The BP of the DEGs was most significantly associated with T cell activation. Significantly enriched immune-related pathways in the KEGG enrichment analysis included Th17 cell differentiation, Th1, and Th2 cell differentiation, and T cell receptor signaling pathway.

### 3.2 WGCNA and module analysis

**Figure 2:**
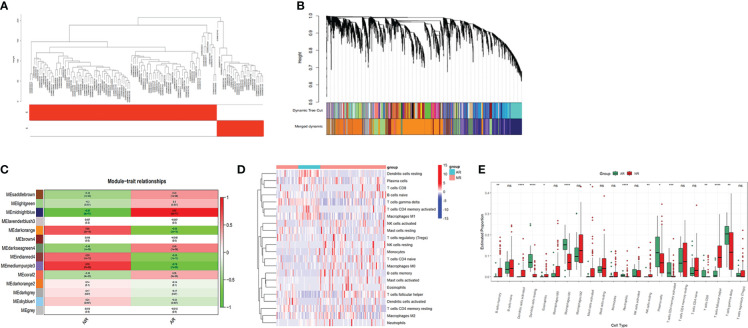
WGCNA and immune cell infiltration analysis of dataset GSE25902: **(A)** Clustering analysis of the samples. **(B)** Gene dendrogram and module colors. **(C)** Module correlation analysis. **(D)** Heatmap for immune cells of the AR and NR samples. **(E)** Comparison of 22 immune cell subtypes between AR and NR groups. *p < 0.05; **p < 0.01; ***p < 0.001; ****p < 0.0001; ns, not significant.

The expression profile of the GSE25902 dataset was downloaded to construct the weighted gene co-expression network using the ‘WGCNA’ R package. The clustering analysis confirmed that there were no outlier samples and two clinical parameters labeled non-rejection and acute rejection to obtain the rejection-related phenotypic traits (**Figure 2A**). To construct a scale-free network, soft threshold was set as β=8, and the correlation coefficient between log(k) and log(p(k)) was greater than 0.9. In total, 13 modules were independently identified, and the grey module indicated genes unable to cluster into any module (**Figure 2B**). Correlations between the modules and the clinical trait are visualized in **Figure 2C**. Based on the rank of their P-Values, two up-regulated modules, mediumpurple3 and dark orange, and one down-regulated module, midnight blue, were selected as rejection-related modules. In these 3 modules, 7235 genes were defined as rejection-related genes according to the criterion GS<0.2 and MM<0.7.

### 3.3 Immune cell infiltration

We uploaded the GSE25902 dataset into CIBERSORTx to analyze the infiltration of 22 types of immune cells. The infiltration results are shown as a heatmap in **Figure 2D**. Comparisons of the immune cell composition between the non-rejection and the acute rejection group were conducted. The acute rejection samples had significantly lower proportions of memory B cells (P<0.01), follicular T cells (P<0.001), resting NK cells (P<0.01), activated Dendritic cells (P<0.001), activated Mast cells (P<0.05) and Eosinophils (P<0.05) than the non-rejection samples. While the proportions of Plasma cells (P<0.05), CD8^+^ T cells (P<0.01), memory activated CD4+ T cells (P<0.001), gamma delta T cells (P<0.01), M1 Macrophages (P<0.001), resting Dendritic cells (P<0.001) and Neutrophils (P<0.001) were significantly higher in the acute rejection group (**Figure 2E**).

### 3.4 Hub gene identification

**Figure 3:**
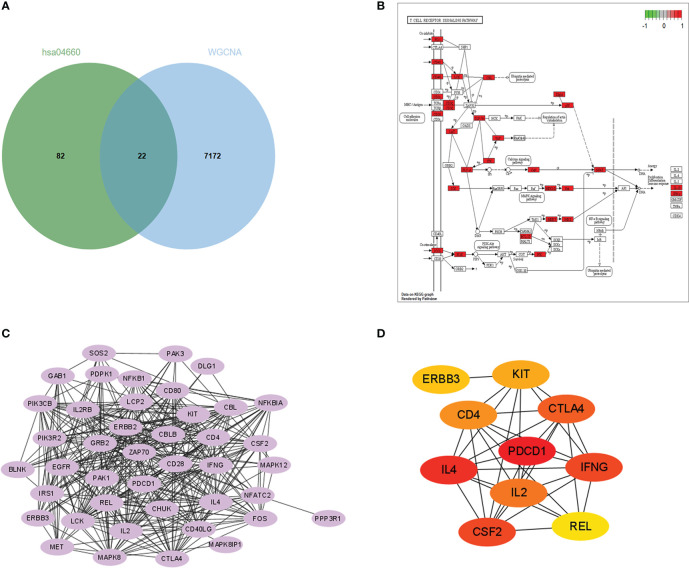
Hub gene identification: **(A)** Venn plot of genes in the midnightblue, mediumpurple3, and darkorange modules and genes in the T cell receptor signaling KEGG pathway (hsa 04660). **(B)** Enrichment of the 22 rejection-related genes in the hsa04660 KEGG pathway. **(C)** PPI network constructed with the 22 rejection-related genes. **(D)** Results of DMNC algorithm from Cytohubba (top 10 genes).

The KEGG enrichment analysis, GO enrichment analysis, and immune cell infiltration analysis all indicated that T cell activation and signaling dominate the mediation of immune response in acute rejection of the renal graft. We selected one significantly enriched KEGG pathway, the T cell receptor signaling pathway (has04660). After overlapping genes in this pathway with the 7194 rejection-related genes from the WGCNA analysis of GSE25902, 22 genes were chosen as rejection-related genes for the next analysis and were annotated into the KEGG diagram (**Figures 3A, B**). PPI network analysis of these genes was performed by STRING and imported into the Cytoscape software (**Figure 3C**). Gene PDCD1 ranked 1 in two (DMNC, ClusteringCoefficient) Cytohubba algorithms (**Figure 3D**). In addition, PDCD1 was also included in the above 1450 intersected DEGs.

### 3.5 Association of hub gene expression with acute rejection

**Figure 4:**
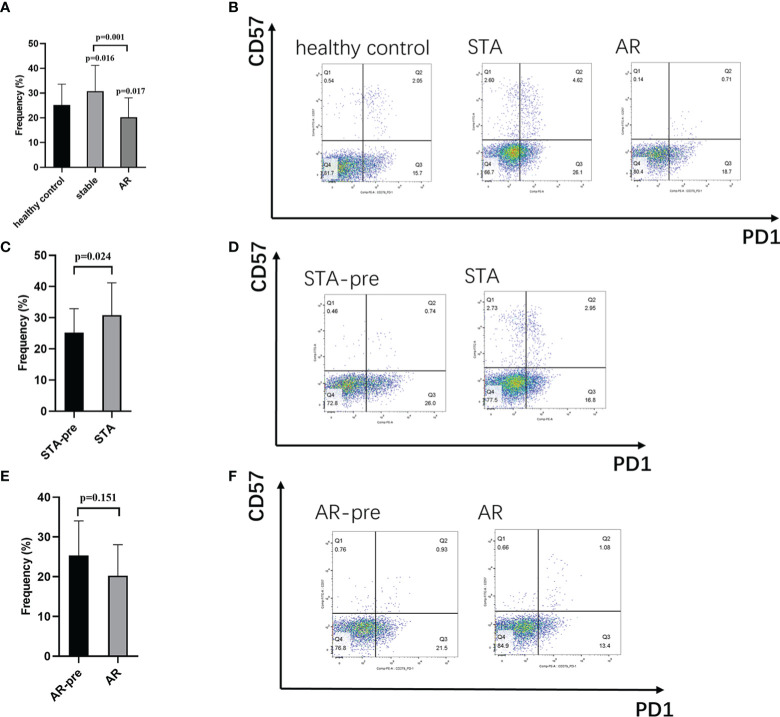
CD4+PD1+CD57- T cells in peripheral blood: **(A)** Recipients with acute rejection (AR) had significantly lower percentages of CD4+PD1+CD57- T cells compared with patients with stable renal function (STA) and the healthy control. **(B)** Representative histograms of CD4+PD1+CD57- T cell expression in the healthy control group, the STA group, and the AR group in flow cytometry. **(C)** Blood samples from the STA recipients had significantly higher percentages of CD4+PD1+CD57- T cells compared with pretransplant blood samples from recipients with stable renal function within 1 year after transplantation (STA-pre). **(D)** Representative histograms of CD4+PD1+CD57- T cell expression in the STA-pre group and the STA group in flow cytometry. **(E)** Blood samples from recipients with AR had significantly higher percentages of CD4+PD1+CD57- T cells compared with preoperative blood samples from those that occurred acute rejection within 1-year after transplantation (AR-pre). **(F)** Representative histograms of CD4+PD1+CD57- T cell expression in the AR-pre samples and the AR in flow cytometry.

**Figure 5:**
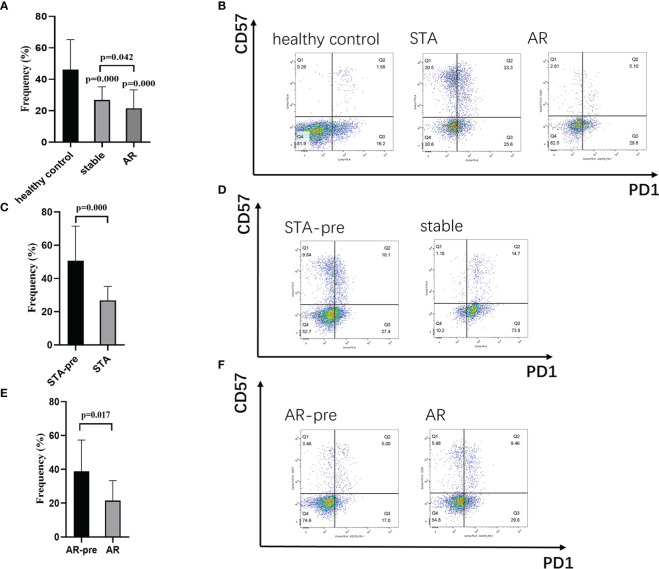
CD8+PD1+CD57- T cells in peripheral blood: **(A)** Recipients with acute rejection (AR) had significantly lower percentages of CD8+PD1+CD57- T cells compared with patients with stable renal function (STA) and the healthy control. **(B)** Representative histograms of CD8+PD1+CD57- T cell expression in the healthy control group, the STA group, and the AR group in flow cytometry. **(C)** Blood samples from the STA recipients had significantly higher percentages of CD8+PD1+CD57- T cells compared with preoperative blood samples from those that remain stable renal function within 1-year after transplantation (STA-pre). **(D)** Representative histograms of CD8+PD1+CD57- T cell expression in the STA-pre group and the STA group in flow cytometry. **(E)** Blood samples from recipients with AR had significantly higher percentages of CD8+PD1+CD57- T cells compared with preoperative blood samples from those occurred acute rejection within 1-year after transplantation (AR-pre). **(F)** Representative histograms of CD8+PD1+CD57- T cell expression in the AR-pre group and the AR group in flow cytometry.

In order to test the expression levels of the identified hub gene, PDCD1, in CD4^+^ and CD8^+^ T cells, whole blood samples from 126 healthy controls, 30 STA recipients, and 12 AR recipients (pre-treatment blood samples), as well as whole blood samples before transplant from 124 recipients showed stable renal function (STA-pre), and 22 recipients occurred acute rejection (AR-pre) within 1 year after transplantation were examined by flow cytometry analysis. The expression levels of CD4^+^PD1^+^CD57^-^ T cells and CD8^+^PD1^+^CD57^-^ T cells in the AR samples were significantly lower than both the STA samples and the healthy control (**Figures 4A, B**, **5A, B**, P<0.05). The STA group showed elevated expression of CD4^+^PD1^+^CD57^-^ T cells and reduced expression of CD8^+^PD1^+^CD57^-^ T cells compared to the STA-pre group (**Figures 4C, D**, **5C, D**, P<0.05). Compared to the AR-pre group, the AR group showed reduced proportions of PD1^+^CD57^-^ phenotypes (P<0.05) in CD8^+^ T cells, while no significant difference was shown in CD4^+^ T cells (**Figures 4E, F**, **5E, F**).

## 4 Discussion

In recent years, acute allograft rejection rates in kidney transplant recipients have dramatically improved with the evolution of immunosuppressive therapy (17). However, prompt differential diagnosis is still essential as acute rejection could rapidly and progressively affect graft function whenever it occurs (18). Identification of biomarkers might provide new insights for early diagnosis or targeted treatment of AR. The current study investigated the KEGG and GO enrichment and the immune cell infiltration of five AR-related GEO datasets from renal biopsy samples, to deeper understand the molecular involvement in AR. We identified 7194 AR-related genes based on WGCNA analysis. By intersection with a specific KEGG pathway and constructing a PPI network, PDCD1, which was then validated with flow-cytometry analysis, was selected as a potential diagnostic marker.

The results of GO biological process terms indicated that the DEGs were involved in T cell activation, mononuclear cell differentiation, regulation of cell-cell adhesion, and lymphocyte differentiation, consistent with the previous enrichment results of TCMR by Zhou et.al (19). The KEGG results mainly enriched the DEGs with multiple autoimmune diseases and various T cell-related pathways, involving differentiation of T helper cells and the T cell receptor signaling pathway. T helper cells, including Th1, Th2, Th17 and follicular helper T cells, act as important immune factors and regulate effective cell pathways in the acute rejection progress (20). The immune infiltration analysis also proved the association of acute rejection with multiple subtypes of T cells, including follicular helper T cells, CD8^+^ T cells, memory-activated CD4^+^ T cells, and gamma delta T cells. Other immune cells, such as B cells, dendritic cells, Neutrophils, Macrophages, etc., were also involved in acute rejection by comparison with non-rejection samples. The participation of these lymphocyte cells was not demonstrated in the enrichment results of the rejection-related DEGs.

In the acute rejection episodes, recognition of the foreign antigen or allograft by T cell receptors generates and mediates alloimmune response by recruiting inflammatory cells or antibodies and destroying the transplant renal function (21–23). Effective T cell activation by T cell receptors would lead to clonal expansion of specific phenotypes of T cells, which can be detected by clinical testing measures (24). Indirect activated CD4^+^ T cells are also capable and unique helpers to alloreactive B cells in the alloantibody-mediated response (25). KEGG is a manually annotated metabolic pathway database that contains highly-ordered functional information on the complex interrelationships between the genes and their products (13). Compared with GO, KEGG is more interactive and more closely linked to the biological progress in reality. For the above reasons, we selected a KEGG enriched pathway, the T cell receptor signaling pathway (hsa04660), from the DEGs-enriched pathways or gene sets for further hub gene selection.

The co-stimulatory and co-inhibitory receptors are have a pivotal role in the T cell receptor signaling, as they modulate the throughout T cell biology, including activation, subset differentiation, effector function, memory formation, and survival (26). Immune checkpoint inhibitors targeting co-stimulatory signals in anti-cancer settings have long been heavily adopted forms of immunosuppressants (27). Knowledge of costimulatory blockade agents in transplantation tolerance covers not only initial T cell activation but is also gradually extending to multiple steps in alloantibody production (28). Leibler C et al. suggested that Belatacept, a recombined CTLA4-Ig, could inhibit the Tfh (PD1^+^ICOS^+^)–B-cell crosstalk in humans and decrease differentiation of B cells into plasmablasts (29). Its direct effects on Ig production by activated B cells are independent of T-cell intervention (28). Therefore, identification of a pivotal biomarker in the T cell receptor pathway, especially a co-stimulatory or co-inhibitory receptor by bioinformatics methods, might provide clues for developing new immunotherapy effective in both T cell- and antibody-related rejections.

PDCD1, which encodes programmed cell death protein 1 (CD279, PD1), is a transmembrane receptor protein in CD28/CTLA-4 subfamily and is broadly expressed in various hematopoietic cells and other tissues (30). There have been great concerns about PD1-related pathways in the induction and maintenance of immune tolerance in cancer, autoimmune diseases, and chronic infection (30). Antibodies blocking the PD1/PDL1 axis have been developed to protect T cells from exhausted status in anti-cancer immunity (31). However, previous studies of PD1 expression in allograft rejection showed conflicting results. Studies by Wang and Afaneh C et al. reported higher levels of PD1 mRNA before renal transplantation and at rejection in both blood and urine samples, which were also associated with disease prognosis (32, 33). While another study by Carmona-Escamilla et al. showed that PD1 was downregulated in CD4^+^ T cells in acute rejection renal recipients (34). PD1 expressions on CD4 and CD8 T cells were also significantly decreased in acute rejection recipients in a previous liver transplantation research (35). Anti-PD1/PDL1 treatments for malignant or metastatic tumors were reported associating with immune checkpoint inhibitors (ICI)-associated transplant rejection. Most of them were acute cellular rejections, and a small proportion was also presented as antibody-mediated rejections (36). The association between PD1 and acute rejection in kidney transplantation might be because of its negative regulations of multiple T cell subsets and humoral immunity (30). Its wide expression in diverse immune cells and tissues might also bring higher diagnostic values and new therapeutic insights, although no agonists of PD1 have yet been available for graft acceptance.

In our flow-cytometry results, acute renal transplant rejection recipients present a significant decrease of the PD1^+^CD57^-^ phenotype in both CD4^+^ and CD8^+^ T cells. PD1^+^CD57^-^ has been recognized as an exhausted T cell phenotype, which refers to reduced capacity to secrete cytokines and expression of inhibitory receptors (37). Clinical and experimental evidence have supported the protective role of exhausted lymphocyte cells in organ transplantation (38). A previous study by Fribourg M et al. showed that increased percentages of PD1^+^CD57^-^ in CD4^+^ and CD8^+^ T cells significantly improved posttransplant renal function and were consistent with the acute rejection incidence (39). We may speculate that monitoring the phenotypic markers, such as PD1^+^CD57^-^, and tapering T-cell depleting induction therapy could effectively reduce acute rejection incidence and immune-related adverse outcomes.

There are several limitations in the present study. Firstly, the sample size for validation was relatively small and restricted to a single center. Secondly, we know that there are two typical classifications of acute rejection, T cell-mediated rejection, and antibody-mediated rejection. We did not respectively investigate each classification. Thirdly, although the PD1^+^CD57^-^ phenotype typically indicates T cell exhaustion, the correlation between CD57 and acute rejection, and interaction between PD1 and CD57 still need to be confirmed.

In conclusion, this study performed comprehensive bioinformatic methods, including WGCNA and immune infiltration analysis. PDCD1 was identified as a diagnostic biomarker in acute renal rejection. The flow-cytometry validation results of the PD1^+^CD57^-^ phenotype are consistent with our hypothesis and suggest the potential role of the PDCD1 gene and the PD1^+^CD57^-^ phenotype in allograft protection. Future research may focus on investigating specific functions of PD1 and the PD1^+^CD57^-^ phenotype in diverse T cell subtypes, such as effector T cell, regulatory T cell, or follicular T cell, as well as B cells, to figure out the underlying mechanisms and to develop new strategies for preserving protective immune responses.

## Data availability statement

The original contributions presented in the study are included in the article/**Supplementary Material**, further inquiries can be directed to the corresponding author/s.

## Ethics statement

The studies involving human participants were reviewed and approved by Clinical research committee of the First Affiliated Hospital, Zhejiang University School of Medicine. Written informed consent for participation was not required for this study in accordance with the national legislation and the institutional requirements.

## Author contributions

YW, XLin: review of public databases, flow cytometry, writing the manuscript. CW: flow cytometry. XLiu, XW, YQ, YC, QZ, HZ: collect clinical samples, overall review of results, data and statistical analysis. JC, HH: supervise the study, funding acquisition, review the manuscript. All authors agree to be accountable for the content of the work.

## Supplementary material

Supplementary Figure 1 Heatmaps for the five selected datasets: **(A–E)** heatmaps for GSE1563, GSE25902, GSE36059, GSE98320, and GSE174020 (demonstrate top 50 DEGs).
