# Supplementary figures and images for: Identification of PDCD1 as a potential biomarker in acute rejection after kidney transplantation via comprehensive bioinformatic analysis
Source: Front Immunol. 2023 Jan 27;13:1076546. doi: 10.3389/fimmu.2022.1076546 (PMC9911868; doi:10.3389/fimmu.2022.1076546)

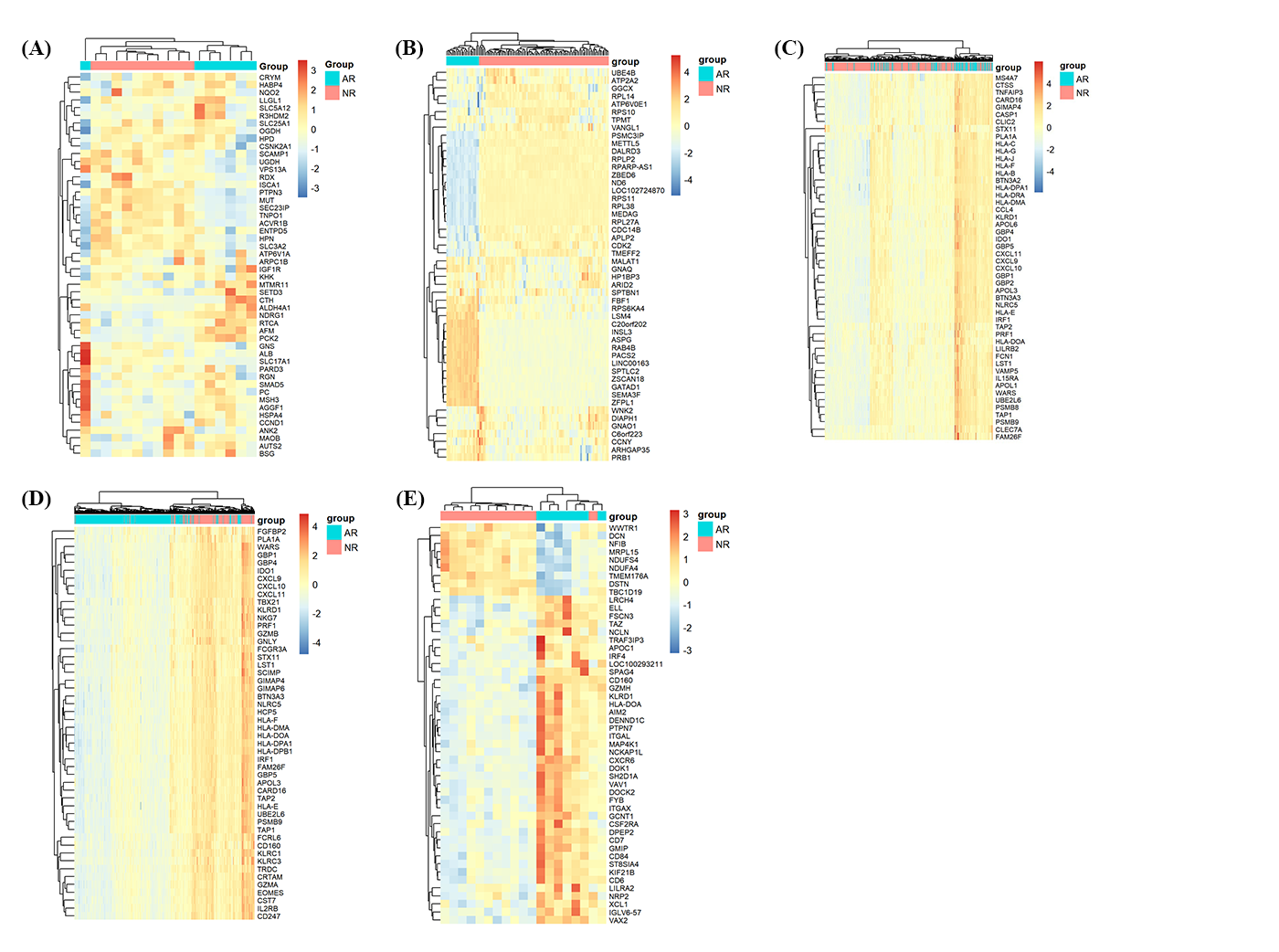

Supplement: Supplementary Figure 1 — Heatmaps for the five selected datasets: (A–E) heatmaps for GSE1563, GSE25902, GSE36059, GSE98320, and GSE174020 (demonstrate top 50 DEGs). [file Image_1.tif]
